# Vitamin D regulating TGF-β induced epithelial-mesenchymal transition

**DOI:** 10.1186/s12931-014-0146-6

**Published:** 2014-11-21

**Authors:** Kimberly D Fischer, Devendra K Agrawal

**Affiliations:** Department of Medical Microbiology and Immunology, Creighton University School of Medicine, Omaha, Nebraska USA; Center for Clinical and Translational Science Creighton University School of Medicine, CRISS II Room 510, 2500 California Plaza, Omaha, NE 68178 USA

## Abstract

**Background:**

Subepithelial fibrosis is a characteristic hallmark of airway remodeling in asthma. A critical regulator of fibrosis, transforming growth factor β (TGF-β), can induce airway remodeling in epithelial cells through induction of epithelial-mesenchymal transition (EMT). Vitamin D has immunomodulatory functions, however, its effect on controlling subepithelial fibrosis is not known.

**Methods:**

Human bronchial epithelial cells (BEAS-2B) were exposed to calcitriol followed by stimulation with TGF-β1 or TGF-β2. The protein expression and mRNA transcripts for E-cadherin, Snail, vimentin, and N-cadherin were analyzed by Western blot and qPCR. An invasion assay and scratch wound assay were performed to identify the migratory properties of the cells following treatments.

**Results:**

TGF-β1 decreased E-cadherin expression and increased protein expression and mRNA transcripts of Snail, vimentin, and N-cadherin together with increased cell invasion and migration. TGF-β2 elicited migratory response similar to TGF-β1 but induced the expression of EMT markers differently from that by TGF-β1. Calcitriol attenuated TGF-β1- and TGF-β2-induced cell motility. Also, calcitriol inhibited the expression of EMT markers in TGF-β1-treated epithelial cells with less effect on TGF-β2.

**Conclusions:**

These data suggest that calcitriol inhibits both migration and invasion induced by TGF-β1 and TGF-β2 in human airway epithelial cells. However, the regulatory effect of vitamin D in epithelial-mesenchymal transition was more effective to TGF-β1-induced changes. Thus, calcitriol could be a potential therapeutic agent in the prevention and management of subepithelial fibrosis and airway remodeling.

## Background

Asthma afflicts more than 300 million people worldwide and is one of the most common chronic disorders of childhood that affects an estimated 6.2 million children under the age of 18 [1]. Asthma is a chronic inflammatory disease that results in the narrowing of the airways, tightening of the chest, shortness of breath, and coughing. The hallmarks of asthma include airway obstruction, chronic wheezing, airway hyperresponsiveness, airway remodeling, inflammation, and mucus hypersecretion. While current treatments include corticosteroids, leukotriene antagonists, and long-acting β2 agonists, these therapies are not effective in preventing or reversing airway remodeling in patients suffering from chronic allergic asthma [2]. In addition, the beneficial anti-inflammatory effect of corticosteroids is not without many adverse effects. Therefore, further understanding of the mechanisms underlying airway remodeling is required to develop therapies that target the molecules involved in structural changes, including fibrosis and epithelial thickening.

Recently, vitamin D has received more attention as an effective immunomodulator in extra-musculoskeletal tissues. Vitamin D is a steroid hormone that is synthesized from cholesterol in the skin, or can be ingested through dietary sources. Vitamin D goes through sequential hydroxylation steps in the liver and kidney resulting in its final active form, 1,25(OH)_2_D_3_ or calcitriol. Calcitriol regulates bone, calcium, and phosphate metabolism through vitamin D receptor (VDR). The VDR forms a heterodimer with the retinoid X receptor and regulates gene expression in the nucleus. The 1,25(OH)_2_D_3_ can also bind to the VDR on the plasma membrane to exert rapid responses via production of second messengers [3]. The increased incidence of asthma [4] associated with increased vitamin D deficiency might suggest a link between the two in the pathogenesis of asthma [5-7]. Previous studies suggest that vitamin D status is a strong predictor of childhood asthma, with deficiency more frequent in children suffering from asthma compared to non-asthmatic controls [8,9]. This association of vitamin D deficiency and asthma is not limited to children and includes prospective studies that suggest vitamin D insufficiency and deficiency are linked with severe and uncontrolled adult asthma [10]. While this data suggests that vitamin D deficiency results in an increased risk for asthma and allergy, the amount of vitamin D that might be required to prevent or lessen the severity of an asthma attack is still unknown. A prospective study found that vitamin D supplementation in asthmatic children prevented asthma exacerbation triggered by acute respiratory infection [11]. However, other reports do not suggest a role for vitamin D supplementation. In two recent studies vitamin D supplementation in asthma patients did not result in significant difference compared to the placebo group [12,13].

Airway remodeling, a consequence of long-standing asthma, reduces lung function and is not regulated well with current therapies. There is currently limited information on the role of vitamin D as a potential inhibitor of airway remodeling in asthma. Previous work has demonstrated that vitamin D plays a role in airway smooth muscle, but the role of vitamin D in the epithelium is not well understood [14,15]. One of the key cytokines involved in the airway remodeling process is TGF-β, which is released from degranulated eosinophils and mast cells to induce multiple localized effects resulting in airway remodeling [16-18].

Airway epithelial cells are located between the host and external environment, designating their key role in the protection of the host against microorganisms, dust, and other allergens. TGF-β has been suggested to initiate fibrosis in the airway epithelial cell through activation of epithelial mesenchymal transition (EMT) signals [19,20]. One of the main transcription factors involved in this process is Snail. It has been demonstrated that Snail forms a transcriptional repressor with Smad3 and Smad4 to promote EMT [21]. Activation of EMT signaling permits the cells to differentiate into myofibroblasts, enabling invasion and migration outside of the epithelium. Increased myofibroblasts in the submucosa secrete collagen and extracellular matrix, thereby, contributing to the subepithelial fibrosis in airway remodeling [22].

While vitamin D has been shown to have immunomodulatory properties in immune cells [10] and airway smooth muscle cells [15], the properties of vitamin D in epithelial cells are currently lacking. Here, we investigated the effect of active form of vitamin D, calcitriol, and TGF-β on EMT markers in bronchial epithelial cells. While most studies examining EMT *in vitro* utilize TGF-β1, there is little information on the effect of TGF-β2, which has also been identified as a contributor to the development of asthma [23,24]. Our findings suggest that calcitriol can inhibit epithelial cell motility induced by both TGF-β isoforms, but calcitriol-induced inhibition of EMT characteristics in response to TGF-β1 and TGF-β2 could involve different molecular mechanisms.

## Methods

### Cell culture

Bronchial epithelial cells, BEAS-2B cells (ATCC, Manassas, VA) were seeded on 6-well culture dishes at 1.5 × 10^5^ cells/ml and cultured in BepiCM media (ScienCell, Carlsbad, CA) supplemented with 10% FBS. When cells reached 50% confluence, the media was changed and cells received serum-free media for 24 hours. Cells were then subjected to stimulation with either 0.1% 95% ethanol (vehicle) or 100 nM calcitriol (Sigma-Aldrich, St. Louis, MO) for 24 hours. TGF-β1 or TGF-β2 (PeproTech, Rocky Hill, NJ) were then added to the cells for an additional 48 hours.

### RNA isolation and qPCR

Total RNA was isolated using the RNeasy Plus Mini kit (Qiagen, Valencia, CA) according to manufacturer’s instructions. RNA concentration was quantified using a Nanodrop (Thermo‐Scientific, Rockford, IL). First-strand cDNA synthesis was performed using 1 μg total RNA with oligo dT, 5X reaction buffer, MgCl2, dNTP mix, RNAse inhibitor and Improm II reverse transcriptase as per manufacturer’s instructions in the Improm II reverse transcription kit (Promega, Madison, WI). Following the first strand synthesis, real time PCR was performed using 3.2 ng of cDNA, 10 μl SYBR Green PCR Master Mix (BioRad Laboratories, Hercules, CA) and forward and reverse primers (10 pmol/μl) (Integrated DNA Technologies, Coralville, IA) using a real time PCR system (CFX96, BioRad Laboratories, Hercules, CA). Relative mRNA transcripts levels were normalized against an internal housekeeping gene and sample differences determined using ^ΔΔ^Ct relative method. A complete list of the primers and their sequences is provided in Table 1.

**Table 1:** **Primer sequences used for qPCR experiments**

**Gene**	**Sequence**	
E-cadherin	Forward	AAG AAG CTG GCT GAC ATG TAC GGA
	Reverse	CCA CCA GCA ACG TGA TTT CTG CAT
Snail	Forward	TTT CTG GTT CTG TGT CCT CTG CCT
	Reverse	TGA GTC TGT CAG CCT TTG TCC TGT
MMP2	Forward	AGA AGG ATG GCA AGT ACG GCT TCT
	Reverse	AGT GGT GCA GCT GTC ATA GGA TGT
MMP9	Forward	ATT TCT GCC AGG ACC GCT TCT ACT
	Reverse	CAG TTT GTA TCC GGC AAA CTG GCT
18S	Forward	TCA ACT TTC GAT GGT AGT CGC CGT
	Reverse	TCC TTG GAT GTG GTA GCC GTT TCT
GAPDH	Forward	TCG ACA GTC AGC CGC ATC TTC TTT
	Reverse	ACC AAA TCG GTT GAC TCC GAC CTT
N-cadherin	Forward	TGT GGG AAT CCG ACG AAT GGA TGA
	Reverse	TGG AGC CAC TGC CTT CAT AGT CAA
Vimentin	Forward	AGA ACC TGC AGG AGG CAG AAG AAT
	Reverse	TTC CAT TTC ACG CAT CTG GCG TT

### Western blot

BEAS-2B cells were washed with ice-cold PBS and incubated with 0.15 ml of modified RIPA lysis buffer with protease and phosphatase inhibitor cocktails 1 and 2 (Sigma-Aldrich, St. Louis, MO). Protein concentration was determined by the BCA protein assay kit (Sigma-Aldrich, St. Louis, MO) according to the manufacturer’s instructions. Each sample of protein containing 15 μg of protein was mixed with equal volume of Laemmli buffer containing 10% 2-mercaptoethanol. Proteins were resolved on 10-20% polyacrylamide gel (BioRad, Hercules, CA). Proteins were transferred onto a nitrocellulose membrane (BioRad, Hercules, CA), which were subsequently blocked with 5% non-fat dry milk for one hour. The membrane was then incubated overnight at 4°C with E-cadherin (ab15148, Abcam, Cambridge, MA), Snail (NBP1-19529, Novus, Littleton, CO), and Vimentin (sc-6260, Santa Cruz**,** Dallas, Texas). Following, a horseradish peroxidase-conjugated secondary antibody (Novus, Littleton, CO) was added to the membrane. The protein expression was detected by ECL chemiluminescence detection reagents (Bio-Rad, Hercules, CA). The immunoreactivity was captured by the ChemiDoc™ MP System (Bio-Rad Laboratories, Hercules, CA). Membranes were stripped and reprobed for N-cadherin and GAPDH. Results were normalized against housekeeping gene GAPDH.

### Zymography

Supernatant from the stimulated BEAS-2B cells were collected, centrifuged to remove debris, and subjected to gelatin zymography. An equal volume of conditioned media from each sample was then run on an 8% SDS-PAGE containing gelatin (1.0 mg/ml). Following electrophoresis, the gels were washed in Triton X-100 and incubated for 18 hours in 50 mM Tris–HCl buffer containing 0.2 mol/L NaCl and 10 mmol/L CaCl_2_. Gels were stained with Brilliant Blue R250 and destained. MMP activity was assessed by observing white bands against a dark background.

### Invasion assay

Following stimulation with calcitriol and/or TGF-β1/TGF-β2, BEAS-2B cells were isolated and reseeded onto 250 μg/ml of growth factor reduced Matrigel™ coated transwell inserts at 1.5 × 10^5^ cells/ml. Supernatant from these cells was used as the chemoattractant. After 48 hours, cells on the top of the inserts were removed and the remaining cells on the bottom were fixed and stained with the Diff-Quick stain kit (Fisher Scientific, Waltham, MA). The density of the invasive cells on the bottom of the insert was examined by counting the cells in five fields per insert locations under a light microscope at 20x magnification.

### Scratch wound healing assay

BEAS-2B cells were seeded, allowed to reach 75% confluency, and serum starved for 24 hours and subsequently stimulated with 100 nM calcitriol for 24 hr. A wound line was generated by using a sterile 10 μl pipette tip followed by the addition of 10 ng/ml TGF-β1 or TGF-β2 for an additional 48 hours. Images were obtained at 0 and 48 hours using a light microscope at 20x magnification with a digital camera under bright field illumination using an Olympus CKX41 microscope.

The distance between the edges of the wound was measured at six different areas from the wound edge-to-edge using ImageJ software. The area between the wound edges was measured at each time point using ImageJ software (as described previously by Dr. Kees Straatman, Advanced Imaging Facilities, University of Leicester, Leicester, UK). The measurements were then converted into a percentage using the formula: % of wound closure = (measurement at 48 h/measurement at time 0 h) * 100; then to obtain the % of wound closure: 100% - % of wound remaining [25].

### Data analysis

Data is expressed as mean ± SEM from three or four independent experiments. Multiple group comparison was performed using one-way analysis of variance with a Dunnett or Tukey post–hoc test. GraphPad Prism v5.0 was used to analyse data with a *p* value of <0.05 considered significant.

## Results

### TGF-β1 induces EMT characteristics

**Figure 1:**
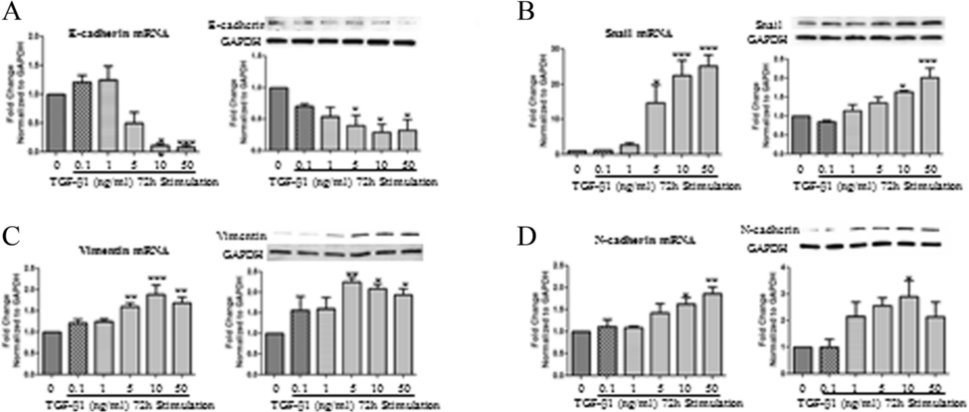
**mRNA and protein expression changes in BEAS-2B cells upon TGF-β1 dose response treatment.** BEAS-2B cells were stimulated with increasing doses of TGF-β1 starting from 0.1 ng/ml and up to 50 ng/ml for 72 hours. Total RNA and protein were isolated and assessed for the expression of **A**: E-cadherin, **B**: Snail, **C**: Vimentin, **D**: N-cadherin, and by means of quantitative real-time PCR and western blot respectively. Protein and mRNA expression levels were normalized to the housekeeping GAPDH and calculated as mean level of induction in comparison to control untreated cells. Data is presented as mean ± SEM n =4, *p <0.05, **p <0.01, and ***p <0.001 by one-way ANOVA.

EMT is defined by changes in gene expression in which epithelial markers such as E-cadherin decrease while mesenchymal markers such as N-cadherin and vimentin increase. BEAS-2B cells were stimulated with increasing doses of TGF-β1 ranging from 0.1 ng/ml up 50 ng/ml. E-cadherin, Snail, vimentin, and N-cadherin mRNA and protein expression were quantified by real time qPCR and Western blotting, respectively. TGF-β1 significantly reduced E-cadherin mRNA at 10 ng/ml and 50 ng/ml and protein levels at 5, 10, and 50 ng/ml (Figure 1A). A significant increase in Snail mRNA expression was observed at 5, 10, and 50 ng/ml and an increase in protein expression at 10 and 50 ng/ml (Figure 1B). A significant increase in vimentin mRNA expression was detected at 5, 10, and 50 ng/ml in which a significant increase in protein expression was observed following treatment with 5, 10, and 50 ng/ml of TGF-β1 (Figure 1C). A significant increase in N-cadherin mRNA expression was noted at 10 ng/ml and 50 ng/ml. This was associated with a significant increase in N-cadherin protein expression observed at 10 ng/ml (Figure 1D). Based on these observations, a concentration of 10 ng/ml was chosen for future experiments.

**Figure 2:**
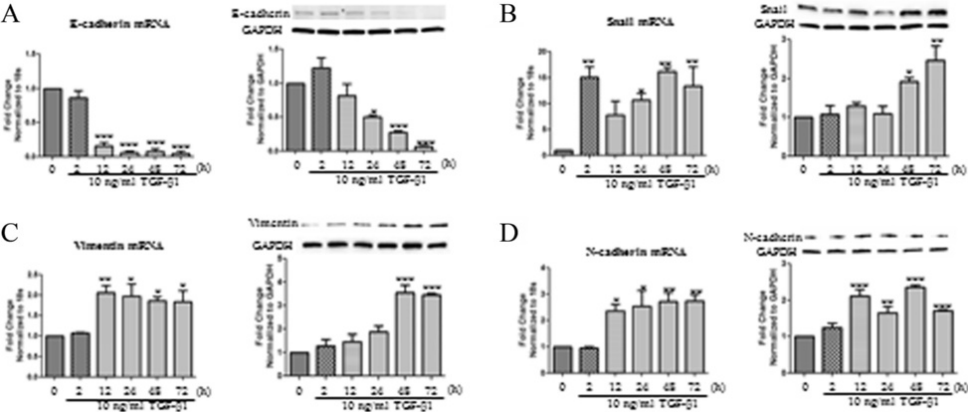
**mRNA and protein expression changes in BEAS-2B cells upon TGF-β1 time course.** BEAS-2B cells were stimulated with 10 ng/ml of TGF-β1 at 72, 48, 24, 12, and 2 hours. Untreated control cells were used as a control. Total RNA was isolated and assessed for the expression of **A**: E-cadherin, **B**: Snail, **C**: Vimentin, **D**: N-cadherin, and by means of quantitative real-time PCR. Expression levels were normalized to the housekeeping 18 s and calculated as mean level of induction in comparison to control untreated cells. Data is presented as mean ± SEM n =3, *p <0.05, **p <0.01, and ***p <0.001 by one-way ANOVA. Total protein was isolated and assessed for the expression of E-cadherin, Snail, N-cadherin, Vimentin, and GAPDH by means of western blot analysis. Expression levels were normalized to the housekeeping gene GAPDH and calculated as mean level of induction in comparison to control untreated cells. Data is presented as mean ± SEM n =4, *p <0.05, **p <0.01, and ***p <0.001 by one-way ANOVA.

Time course responses to TGF-β1 were tested as well on the markers of EMT. Significant decrease in E-cadherin mRNA was observed at 12, 24, 48, and 72 hours while significant changes in E-cadherin protein expression were found at 48 and 72 hours post TGF-β1 stimulation (Figure 2A). Increase in Snail mRNA transcripts was observed 2, 24, 48, and 72 hours post TGF-β1 stimulation. Noteworthy increases in Snail protein expression were noticed at 48 and 72 hours post TGF-β1 stimulation (Figure 2B). Vimentin mRNA expression was increased at 12, 24, 48, and 72 hours while the increase in vimentin protein was prominent at 48 and 72 hours only (Figure 2C). Significant increases in N-cadherin mRNA transcript levels were seen at 12, 24, 48, and 72 hours, while increase in N-cadherin protein levels were observed at 12, 24, 48, and 72 hours (Figure 2D). Based on these findings, a time of 48 hours was chosen for subsequent experiments.

### Calcitriol induces E-cadherin mRNA expression changes in BEAS-2B cells

**Figure 3:**
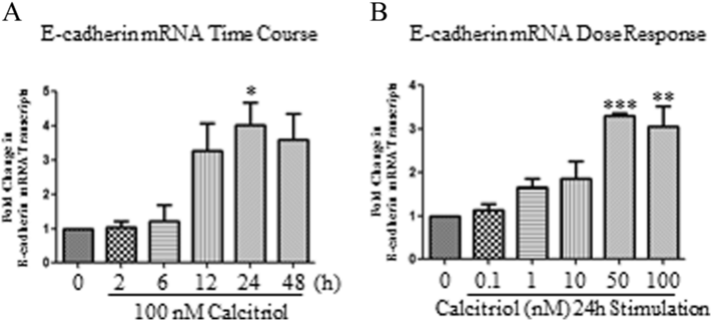
**mRNA expression changes in BEAS-2B cells upon calcitriol time course and dose response.** Calcitriol (100 nM) was added to BEAS-2B cells for 2, 6, 12, 24 and 48 hours. Total RNA was isolated and analyzed for the expression of E-cadherin by qPCR. Expression levels were normalized to the housekeeping gene 18 s and calculated as mean level of induction in comparison to control untreated cells **(A)**. Data is presented as mean ± SEM n =3, *p <0.05, by one-way ANOVA. For calcitriol dose response, response treatment BEAS-2B cells were stimulated with increasing doses of calcitriol starting from 0.1 nM and up to 100 nM for 24 hours. Total RNA and protein were isolated and assessed for the expression of E-cadherin **(B)**. Data is presented as ± SEM n =3, *p <0.05, **p <0.01 one-way ANOVA.

BEAS-2B cells stimulated with calcitriol increased E-cadherin mRNA transcripts when cells were treated with 100 nM calcitriol from 2 to 48 hours, with a significant increase observed at 24 hours (Figure 3A). E-cadherin mRNA levels were then detected following treatment with increasing doses of calcitriol following a 24 hours stimulation. Significant increases were noted at 50 nM and 100 nM of calcitriol (Figure 3B).

### Morphological changes induced by TGF-β1, TGF-β2, and calcitriol

**Figure 4:**
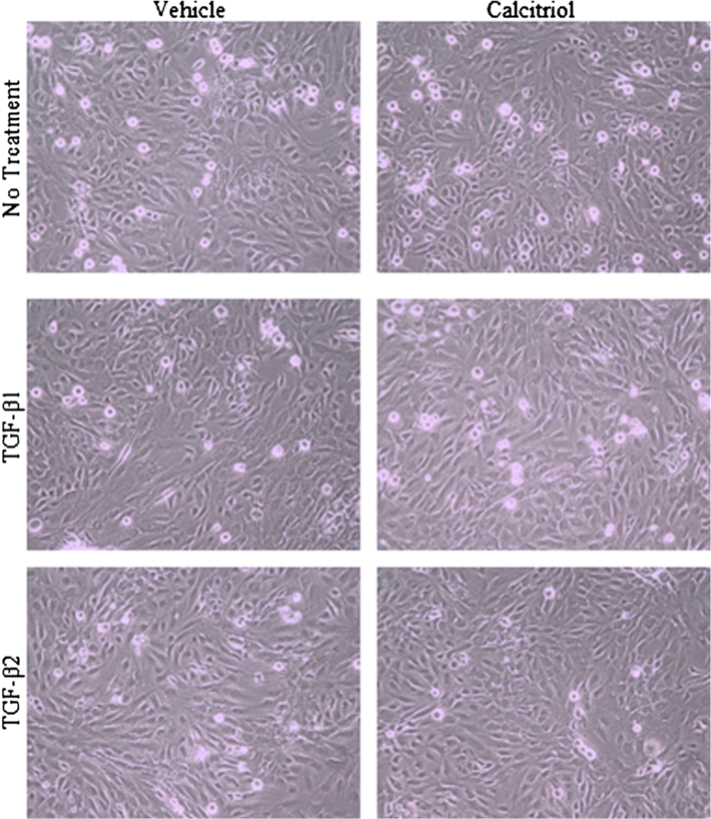
**Morphological changes induced by TGF-β1, TGF-β2, and Calcitriol.** BEAS-2B cells were treated with 0.1% vehicle or 100 nM calcitriol for 24 hours. 10 ng/ml of TGF-β1 or 10 ng/ml of TGF-β2 was added for an additional 48 hours. Pictures were taken with bright field illumination using an Olympus CKX41 microscope.

Stimulation with TGF-β1 and TGF-β2 induced morphological changes in BEAS-2B cells consistent with EMT (Figure 4). Cells stimulated with either TGF-β1 or TGF-β2 developed an elongated, spindle fibroblast-like morphology with reduced cell-cell contact (Figure 4). Such changes in the morphology of the cells were prevented by the addition of calcitriol prior to the stimulation with TGF-β1 and TGF-β2. The morphology of the cells in the presence of calcitriol was similar to that in the control cells maintaining the typical epithelial cobblestone pattern.

### Calcitriol regulates TGF-β-mediated EMT mRNA and protein expression changes in BEAS-2B cells

**Figure 5:**
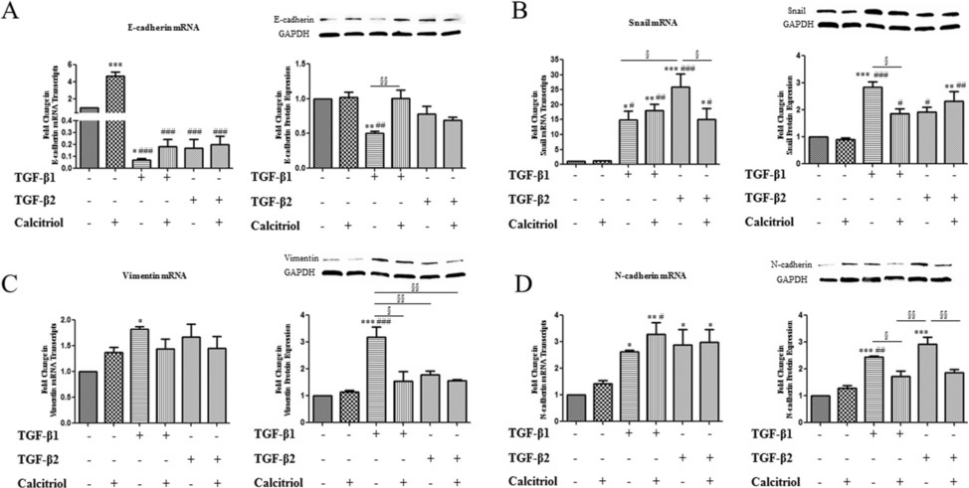
**Calcitriol regulates TGF-β-mediated EMT mRNA and protein expression changes in BEAS-2B cells.** BEAS-2B cells were stimulated with the active form of Vitamin D, calcitriol (100 nM). After 24 hours, TGF-β1 (10 ng/ml) or TGF-β2 (10 ng/ml) was added to the cells for an additional 48 hours. Total RNA was isolated and assessed for the expression of **A**: E-cadherin, **B**: Snail, **C**: Vimentin, **D**: N-cadherin (left panels) by means of quantitative real-time PCR. Expression levels were normalized to the housekeeping 18 s and calculated as mean level of induction in comparison to 0.1% vehicle control cells. Data is presented as mean ± SEM n =4. Total protein was isolated and assessed for the expression of **A**: E-cadherin, **B**: Snail, **C**: Vimentin, **D**: N-cadherin (right panels), and GAPDH by means of western blot analysis. Expression levels were normalized to the housekeeping gene GAPDH and calculated as mean level of induction in comparison to 0.1% vehicle control cells. Data is presented as mean ± SEM n =3. Both qRT-PCR and western blot analysis were analyzed by one-way ANOVA *p <0.05, **p <0.01, and ***p <0.001 compared to control, #p <0.05, ##p <0.01, and ###p <0.001 compared to calcitriol, §p <0.05, §§p <0.01, and §§§p <0.001.

Calcitriol has been shown to be a potential inhibitor of fibrosis [26]. Here, we addressed the potential of calcitriol to prevent the actions of TGF-β1 or TGF-β2 as determined by EMT markers. BEAS-2B cells were pretreated with 100 nM calcitriol for 24 hours and subsequently stimulated with TGF-β1 or TGF-β2 for an additional 48 hours. Analysis of the EMT markers determined that a decrease in E-cadherin mRNA was observed after TGF-β1 and TGF-β2 treatment (Figure 5A left panel). Calcitriol prevented TGF-β1-mediated decreases in E-cadherin protein expression as E-cadherin protein levels were kept at basal levels (Figure 5A right panel). Snail protein expression was increased following TGF-β1 stimulation that was impeded by the presence of calcitriol (Figure 5B right panel). This was not reflected in the mRNA data for Snail, however TGF-β2 increased Snail mRNA transcript levels, which was inhibited by calcitriol treatment (Figure 5B left panel). While no significant TGF-β1 mediated changes in vimentin or N-cadherin were observed in the mRNA (Figures 5C and 5D left panel), significant decreases in vimentin and N-cadherin were found following calcitriol pretreatment and subsequent TGF-β1 stimulation (Figures 5C and 5D right panel). Calcitriol also prevented the TGF-β2 mediated increase in N-cadherin protein expression (Figure 5D right panel).

### Calcitriol prevents TGF-β-mediated EMT: MMP2 and MMP9 mRNA and protein expression changes in BEAS-2B cells

**Figure 6:**
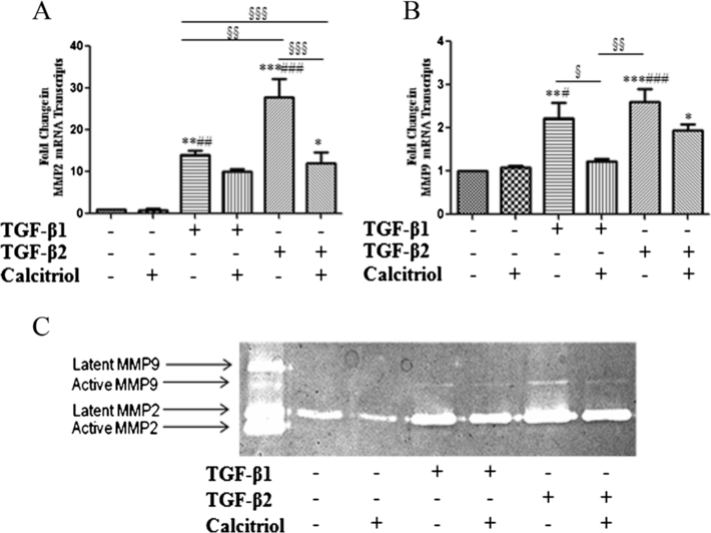
**Calcitriol prevents TGF-β-mediated EMT: MMP2 and MMP9 mRNA and protein expression changes in BEAS-2B cells.** BEAS-2B cells were stimulated with the active form of Vitamin D, calcitriol (100 nM). After 24 hours, TGF-β1 (10 ng/ml) or TGF-β2 (10 ng/ml) was added to the cells for an additional 48 hours. Total RNA was isolated and assessed for the expression of **A**: MMP2 (n =4) and **B**: MMP9 (n =4) by means of quantitative real-time PCR. Expression levels were normalized to the housekeeping 18 s and calculated as mean level of induction in comparison to 0.1% vehicle control cells. Data is presented as mean ± SEM *p <0.05, **p <0.01, and ***p <0.001 compared to control, #p <0.05, ##p <0.01, and ###p <0.001 compared to calcitriol, §p <0.05, §§p <0.01, and §§§p <0.001 by one-way ANOVA. **C**: Conditioned supernatant of BEAS-2B cells stimulated with TGF-β1, TGF-β2 and/or 100 nM calcitriol were subject to gelatin zymography. Results are representative of 3 separate experiments.

EMT has been demonstrated to be involved cellular migration and invasion, processes directed by MMPs. Treatment of BEAS-2B cells with TGF-β1 and TGF-β2 resulted in a significant increase in MMP2 and MMP9 mRNA expression (Figure 6A and Figure 6B). Pretreatment with calcitriol prevented TGF-β2 facilitated increase in MMP2 expression (Figure 6A) and TGF-β1 mediated increase in MMP9 (Figure 6B). The activity of the MMPs was detected using gelatin zymography. Supernatants from TGF-β1 and TGF-β2 stimulated cells show an upregulated pro-MMP2 protein (latent MMP2) and an increase in active MMP9 (Figure 6C).

### Calcitriol inhibits TGF-β-induced invasiveness and migration of BEAS-2B cells

**Figure 7:**
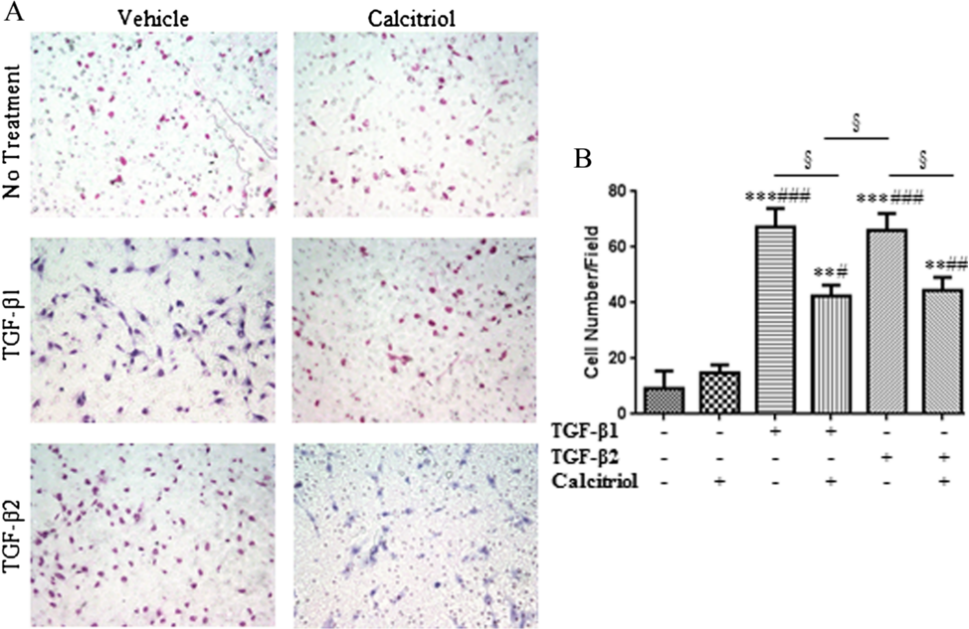
**Calcitriol inhibits TGF-β-induced invasiveness of BEAS-2B cells.** BEAS-2B cells were stimulated with 100 nM calcitriol for 24 hours followed by 48 hours of stimulation by 10 ng/ml of TGF-β1 or TGF-β2. BEAS-2B cells were isolated and reseeded onto 250 ug/ml of growth factor reduced Matrigel™ coated transwell inserts at 1.5 × 10^5^ cells/ml. Conditioned supernatant from these cells was used as the chemoattractant. After 48 hours, cells on the top of the inserts were removed and the remaining cells on the bottom were fixed and stained with the Diff-Quick stain kit. **A**: Representative images of BEAS-2B cells which had migrated through the inserts in response to TGF-β treatment. **B**: Data represent an average of cells counted in 5 random fields. Data is presented as mean ± SEM (n =4), **p <0.01 and ***p <0.001 compared to control, #p <0.05, p## <0.01, and ###p <0.001 compared to calcitriol treated cells, and §p <0.05 analyzed by one-way ANOVA.

**Figure 8:**
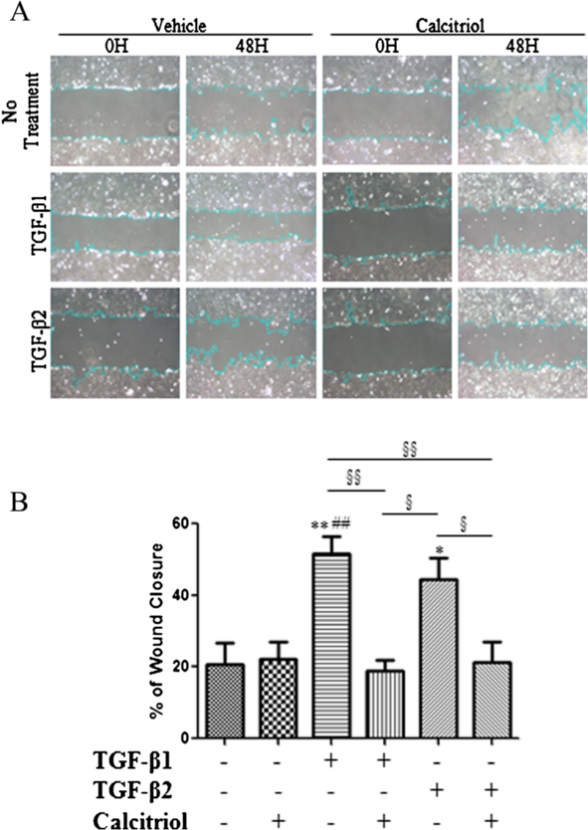
**Calcitriol inhibits the migration of TGF-β stimulated BEAS-2B cells.** BEAS-2B cells were stimulated with 100 nM calcitriol for 24 hours followed by 48 hours of stimulation by 10 ng/ml of TGF-β1 or TGF-β2. **A**: Representative images of a wound healing scratch assay. Pictures of the same area were taken at 0 and 48 hours at 20x magnification. The area of the wound was measured using the NIH ImageJ program. **B**: The % wound closure was calculated and means of groups were compared by one-way ANOVA. Data is presented as mean ± SEM (n =4), *p <0.05 and **p <0.01 compared to control, ##p <0.01 compared to calcitriol treated cells, §p <0.05, §§p <0.01.

To assess the ability of TGF-β-treated BEAS-2B cells to undergo another EMT characteristic, mobility, an invasion assay and scratch wound healing assay were utilized. Matrigel™ coated inserts were removed and the number of cells invading through were counted (representative images Figure 7A). Cells treated with TGF-β1 and TGF-β2 had a 70% increase in invasion compared to unstimulated and calcitriol stimulated BEAS-2B cells. Pretreatment with calcitriol decreased cell invasion to around 40% (Figure 7B). Calcitriol also prevented TGF-β1 and TGF-β2 mediated BEAS-2B cell migration as noted in the scratch wound healing assay (Figure 8A). Pretreatment with calcitriol significantly decreased the effects of TGF-β1 and TGF-β2 by approximately 50% (Figure 8B).

## Discussion

In normal epithelium, injury to the epithelium leads to the release of soluble factors including cytokines, chemokines, prostaglandins, transforming growth factor (TGF), epidermal growth factor (EGF), fibroblast growth factor (FGF), matrix metalloproteinase (MMP), which can all promote cell adhesion junction remodeling and migration during wound repair [27]. Activation of fibroblasts and myofibroblasts is important for wound closure and healing, but may become aberrant in the presence of increased inflammation. Myofibroblasts can originate from different precursor cells including fibroblasts, mesenchymal stem cells, bone marrow-derived mesenchymal stem cells, smooth muscle cells, and epithelial cells which are derived through induction of a process termed epithelial-mesenchymal transition (EMT). Following injury to the epithelium, an epithelial cell will go through architectural rearrangement to enable spreading, migration and secretion of extracellular matrix (ECM) [27,28].

Chronic asthma is characterized by structural changes in the lung termed airway remodeling resulting in a decline in lung function regardless of anti-inflammatory treatment [2]. The airway epithelial cells are at the frontlines of protection in the airway. Following damage from pollutants or allergens, the epithelium can repair itself through initiation of the EMT process. In general, epithelial stress will initiate the generation of mesenchymal cells for tissue generation and tissue closure, with attenuation of EMT following the completion of repair. However, it is thought in the context of inflammation in the asthmatic airway, the release of EMT signaling is not discontinued, but amplified [29]. During the repair process, cell-cell and cell-adhesion contacts are remodeled with loss of the epithelial marker such as E-cadherin and increased expression of mesenchymal markers of vimentin, N-cadherin, and α-smooth muscle actin (α-SMA). Additionally, epithelial cells will also secrete MMPs in response TGF-β1, indicating a possible role in migration of epithelial cells through the basement membrane [30]. These changes in the polarized epithelial cell result in functional changes, allowing the epithelial cells to become motile and degrade the underlying ECM and move below the epithelium. There is evidence to suggest that airway remodeling and hyperresponsiveness in asthma may be driven by repeated exposures to allergens or environmental toxins, leading to the inappropriate repair of airway epithelial cells [31,32].

Eosinophils commonly secrete TGF-β. However other cells including macrophages, lymphocytes, fibroblasts, epithelial cells, and mast cells can also secrete TGF-β [33]. The number of eosinophils found in the asthmatic lung has been found to be increased in bronchial biopsies of asthmatic patients [34] and BAL fluid in mice [35]. One of the major cytokines secreted by eosinophils is that of TGF-β [36]. TGF-β has also been found to be increased in the BAL fluid and tissue of asthmatic patients. However, which isoform is expressed and the tissue location is still debatable. Overall, TGF-β1 [37] and TGF-β2 [24] were found to be in higher amounts in the BAL fluid of asthmatics compared to control patients. Both TGF-β1 and TGF-β2 were found to be expressed by eosinophils and contribute to increase number of eosinophils in the airway [23,36,38]. The pathogenesis of airway remodeling has been thought to be an activation of the epithelial-mesenchymal trophic unit in which increased levels of TGF-β contribute to the activation of epithelial cells to transform into myofibroblasts [39]. While TGF-β1 has been found to be involved in the EMT process, it has not been established whether TGF-β2 has the same effect. Our results here are consistent with previous contributions in that we observe BEAS-2B cells treated with TGF-β1 have increased markers of EMT with a loss of E-cadherin. Conversely, TGF-β2 treatment of BEAS-2B cells presented an expression pattern of EMT markers which was different from that induced by TGF-β1.

Although the contribution of EMT in asthma is still highly debated, there is evidence that epithelial cells contribute to the pool of myofibroblasts [22]. This increase in myofibroblasts may be a part of the airway remodeling process as noted in chronic asthma. TGF-β1 has been demonstrated to be responsible for differentiation into myofibroblasts and this effect was found not be abrogated by corticosteroid treatment [40,41]. Therefore, finding an effective alternative therapy to anti-inflammatory drugs is of great significance. Calcitriol was shown to prevent the effects of EMT in rat lung and mouse lung fibroblasts treated with TGF-β [42]. Anti-inflammatory effect of vitamin D on a steroid resistant gene was also observed in airway smooth muscle cells treated with TNFα and IFNγ [43]. We were, therefore, interested in observing the effects of calcitriol on TGF-β treated BEAS-2B cells. Markers of EMT, such as loss of E-cadherin, increased expression of N-cadherin, and vimentin, were observed at the mRNA and protein level following treatment with TGF-β1. This increase in TGF-β was impeded by pre-stimulation with calcitriol, as indicated by qPCR and Western blot. Our data suggest that both TGF-β1 and TGF-β2 enhance BEAS-2B cell invasion and MMP2 mRNA expression and that this process is abrogated by calcitriol.

A recent study included adult asthma patients on the inhaled ciclesonide and levalbuterol combined with an initial dose of 100,000 IU of vitamin D_3_ followed by a daily dose of 4,000 IU. In this study, there were no differences in the indices of asthma control, asthma attacks, or improved quality of life in adult asthmatics compared to the placebo group. Sputum eosinophilia, lung function, or airway hyperreactivity were also not improved following treatment with Vitamin D_3_ [12]. However, in patients that had responded well to the vitamin D_3_ treatment or had reached a 25-hydroxyvitmain D level of 30 ng/ml or greater, these patients had a lower rate of first exacerbation rates, and lower overall rates of exacerbation and treatment failures [11]. Unfortunately, the authors did not determine if vitamin D treatment modified sputum or blood inflammatory biomarkers. None-the-less, these results support the therapeutic benefit of vitamin D in allergic asthma, perhaps as an adjunct therapy. Another recent study investigated the role of vitamin D supplementation in asthmatic children aged 6–18 years with mild asthma and insufficient vitamin D levels. Following 6 weeks of 2,000 IU of vitamin D daily, there was no difference in the effect of vitamin D compared to placebo on methacholine challenge test, IgE levels, airway cytokines, and eosinophilia although there was a significant increase in serum vitamin D levels. However, the relatively small sample size of 36 patients of this study prevents any discernable conclusions [13].

The Institute of Medicine recommendations for adequate vitamin D intake to maintain 25(OH)D_3_ serum value at 20 ng/ml or 50 nM. However, many clinical laboratories continue to routinely report a value of 20 ng/ml as inadequate and that value 21–29 ng/ml (52.5-72.5 nM) are insufficient [44]. Vitamin D supplemented in the diet of OVA-sensitized mice resulted in a decreased severity of airway remodeling [45]. These mice, supplemented with 10,000 IU/kg or 2,000 IU/kg of vitamin D ended up with serum 25(OH)D levels of 67.13 ng/ml and 31.00 ng/ml, respectively [46]. Vitamin D supplementation reduced airway hyperresponsiveness, airway remodeling, and BALF cytokine levels [45]. However, vitamin D supplementation did not fully reverse the effects of allergic airway inflammation. The physiological range of calcitriol has been observed to be around 0.05 to 0.16 nmol/L. The ability of bronchial epithelial cells to convert inactive vitamin D to its active form has recently been demonstrated, indicating that the local concentration of calcitriol is much higher at the local cellular level [47]. Therefore, to address the difference between sufficient and supplementation values, higher calcitriol levels of 100 nM were used in this study. While the results of clinical trials utilizing vitamin D in asthma patients have inconsistent results, vitamin D supplementation may still prove to be beneficial to those suffering with asthma Additional careful and controlled studies are warranted to address the controversy on the role of vitamin D deficiency in the pathogenesis of asthma.

The differences in mRNA and protein levels following TGF-β1 or TGF-β2 stimulation suggest that the induction of N-cadherin, snail, and vimentin may occur through different receptors. TGF-β binds to heterodimeric type I and type II TGF-β receptors. There are seven type I receptors and five type II receptors, of which the heterodimeric association of these serine/threonine receptors determines the specificity of the ligand signaling [48]. Co-receptors, such as betaglycan, can also modulate TGF-β1 signaling which is imperative for TGF-β2 signaling [45]. The mechanisms mediating EMT may be dependent on Smad when stimulated with TGF-β1. This process may be increased in the presence of cytokines, including TNF-α [30], IL-22 [49], IL-4 and IL-17 [50]. Johnson *et al.* [22] observed increased expression and nuclear translocation of Snail, a transcriptional repressor of E-cadherin and a potent inducer of EMT, in the airway epithelial cells of HDM-exposed mice and increased TGF-β upregulation following allergen challenge. Furthermore, the authors also found increased phosphorylated-Smad3 and Snail1 in TGF-β/EGF-induced EMT [22]. Additionally, deficiency in the PI3K inhibitor phosphatase and tensin homolog (PTEN), has been associated with increased myofibroblast differentiation [51]. Cytokines such as TNF-α could also induce EMT through Snail stabilization and increased migration and invasion of tumor cells. The cytokine IL-6 was recently shown to induce migration of bronchial epithelial cells in PI3K/Akt/GSK-3β/β-catenin dependent manner suggesting that this pathway may be contributing to the mesenchymal population of cells often found in asthma through induction of EMT signals [52,53]. TGF-β1 has also been found to induce nuclear translocation of relA/p65 of NF-κB, inducing NF-kB gene activity and down-regulating PTEN promoter activity and protein expression via NF-kB [54].

The findings in this study suggest that calcitriol prevents the migration and invasion of TGF-β-treated bronchial epithelial cells. The differences in mRNA and protein data (Figure 5) indicate that calcitriol mediates this process through different mechanisms. Calcitriol has been shown to inhibit NF-κB nuclear translocation into human bronchial smooth muscle cells by decreasing importin α3 expression via VDR [15]. VDR expression was found to be decreased in OVA-sensitized and challenged mice fed with a vitamin D deficient diet [55]. Therefore, importins represent a potential target for vitamin D in alleviating allergic immune responses [56]. Also, deletion of VDR in mouse embryonic fibroblasts reduced the levels of NF-κB p65 protein and the activity of translation regulators eIF2α and protein kinase R [57]. Therefore, the underlying mechanisms of vitamin D regulation and EMT and its involvement in airway remodeling in the airway need further elucidation and may be a point of therapeutic interest.

## Conclusions

This data suggests that calcitriol regulates TGF-β1- and TGF-β2-mediated EMT in bronchial epithelial cells. Given the difference in EMT marker expression for TGF-β1 and TGF-β2 treatment, calcitriol may be inhibiting TGF-β1/β2-mediated-migration and invasion by different and yet undefined cellular processes. Calcitriol may also be initiating its effects both at the mRNA transcript and protein levels. Our results indicate a role for bronchial epithelial cells in myofibroblast formation and thereby airway remodeling which may be inhibited by calcitriol.

## Abbreviations

EMT: Epithelial mesenchymal transition
MMP: Matrix-metalloproteinases
