# Erratum to: Vitamin D regulating TGF-β induced epithelial-mesenchymal transition

**DOI:** 10.1186/s12931-015-0301-8

**Published:** 2015-11-10

**Authors:** Kimberly D. Fischer, Devendra K. Agrawal

**Affiliations:** Department of Medical Microbiology and Immunology, Creighton University School of Medicine, Omaha, NE USA; Center for Clinical and Translational Science Creighton University School of Medicine, CRISS II Room 510, 2500 California Plaza, Omaha, 68178 NE USA

## Erratum

After publication of the original article [1], it came to the authors’ attention that Fig. 8, associated with the wound healing assay to show the migration of the cells following scratch, inadvertently placed the same representative images in both experimental groups (TGF-β1 + calcitriol and TGF-β2 + calcitriol) at 0H and 48H to indicate two different treatment groups. The 0H and 48H time point pictures representing TGF-β2 and calcitriol treatment groups were the same pictures used in the images denoting 0H and 48H time points for the TGF-β1 and calcitriol treatment group.

**Fig. 8:**
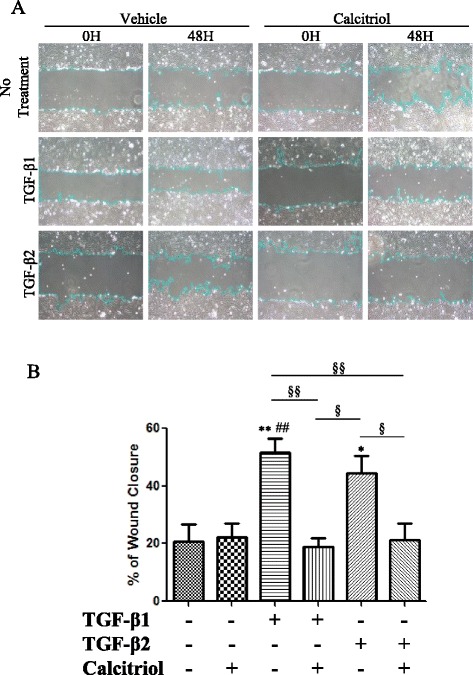
Calcitriol inhibits the migration of TGF-β stimulated BEAS-2B cells. BEAS-2B cells were stimulated with 100 nM calcitriol for 24 h followed by 48 h of stimulation by 10 ng/ml of TGF-β1 or TGF-β2. **a** Representative images of a wound healing scratch assay. Pictures of the same area were taken at 0 and 48 h at 20x magnification. The area of the wound was measured using the NIH ImageJ program. **b** The % wound closure was calculated and means of groups were compared by one-way ANOVA. Data is presented as mean ± SEM (*n* =4), *p <0.05 and **p <0.01 compared to control, ^##^ *p* <0.01 compared to calcitriol treated cells, ^§^ *p* <0.05, ^§§^ *p* <0.01

The image duplication occurred when the images were used as a placeholder for forthcoming data. The choice of image was independent of data analysis and thus does not change the results of this study. The correct image for Fig. 8 is shown below.

The authors sincerely apologize for the inadvertent error and the inconvenience to the journal and the readers.
